# Highly sensitive gas-phase explosive detection by luminescent microporous polymer networks

**DOI:** 10.1038/srep29118

**Published:** 2016-07-04

**Authors:** André Räupke, Alex Palma-Cando, Eugen Shkura, Peter Teckhausen, Andreas Polywka, Patrick Görrn, Ullrich Scherf, Thomas Riedl

**Affiliations:** 1 Institute of Electronic Devices, University of Wuppertal, Rainer-Gruenter-Str. 21, 42119 Wuppertal, Germany; 2 University of Wuppertal, Macromolecular Chemistry, Gaußstr. 20, 42119 Wuppertal, Germany; 3 University of Wuppertal, Chair of Large Area Optoelectronics, Rainer-Gruenter-Str. 21, 42119 Wuppertal, Germany

## Abstract

We propose microporous networks (MPNs) of a light emitting spiro-carbazole based polymer (PSpCz) as luminescent sensor for nitro-aromatic compounds. The MPNs used in this study can be easily synthesized on arbitrarily sized/shaped substrates by simple and low-cost electrochemical deposition. The resulting MPN afford an extremely high specific surface area of 1300 m^2^/g, more than three orders of magnitude higher than that of the thin films of the respective monomer. We demonstrate, that the luminescence of PSpCz is selectively quenched by nitro-aromatic analytes, e.g. nitrobenzene, 2,4-DNT and TNT. In striking contrast to a control sample based on non-porous spiro-carbazole, which does not show any luminescence quenching upon exposure to TNT at levels of 3 ppm and below, the microporous PSpCz shows a clearly detectable response even at TNT concentrations as low as 5 ppb, clearly demonstrating the advantage of microporous films as luminescent sensors for traces of explosive analytes. This level states the vapor pressure of TNT at room temperature.

The highly sensitive and reliable detection of explosives is of paramount importance for civil security. By a similar token, the locating of landmines and bombs as long-term residues of military conflicts is essential to both prevent injuries to humans and to be able to avoid the release of poisonous or carcinogenic explosive chemicals to the environment^1^. Currently, explosive detection either relies on specially trained animals or highly sophisticated measurement techniques, like mass spectrometry, chromatography, Raman spectroscopy, etc.^2^,^3^,^4^. High cost and/or complexity of these techniques limit their wide availability, especially in the field. Compared to that, polymer based explosive sensors seed the prospect of low cost and moderate complexity^5^. At the same time, polymer sensors in which the analyte affects their luminescent properties or their optical gain promise ultra-high sensitivity^6^,^7^,^8^.

Recently, microporous polymer networks (MNPs) have seen a tremendous increase of interest with their applications ranging from energy storage to sensors^9^. For sensing applications, the high surface area of the MNP compared to their dense, non-cross linked, bulk analogues is expected to provide better access of the species to be detected (analyte), which results in an enhanced sensitivity and lower detection levels. Highly sensitive MNP based electrochemical sensors have been demonstrated^10^,^11^. Recently, MNPs have also been considered as luminescent sensors for nitro-aromatic compounds^12^. Surprisingly, the MPNs used in that work showed a strong photoluminescence (PL) response not only to nitro-aromatics but also to benzene and toluene.

While some approaches aim to detect explosives in a liquid environment^13^, the detection of explosives in the gas phase (“sniffing”) would be highly desirable. In this context, 2,4,6-trinitrotoluene (TNT) or 2,4-dinitrotoluene (DNT) have evolved as “fruit-fly” compounds for luminescent explosive sensing due to their relatively high vapor pressures at room temperature. Typically, a wide range of explosive mixtures contain traces of DNT and TNT, which renders these compounds suitable reporters for several explosives^14^.

In this paper, we demonstrate the highly sensitive detection of TNT and DNT vapors by luminescent sensors based on a microporous network of 2,2′,7,7′-tetrakis(carbazol-9-yl)-9,9′spirobifluorene structural units (PSpCz). The PSpCz has been synthesized by electrochemical polymerization from a solution of the monomer SpCz, resulting in luminescent microporous films with a high Brunauer–Emmett–Teller (BET) surface area of 1300 m^2^/g which is more than three orders of magnitude higher than that of a thermally evaporated thin film of the monomer SpCz (0.88 m^2^/g). We demonstrate, that the luminescence of PSpCz is selectively quenched by nitro-aromatic analytes. In striking contrast to a control sample based on thermally evaporated (non-porous) SpCz, which does not show any luminescence quenching upon exposure to TNT at levels of 3  ppm and below, the microporous PSpCz shows a clearly detectable response even at TNT concentrations as low as 5 ppb.

## Results and Discussion

**Figure 1:**
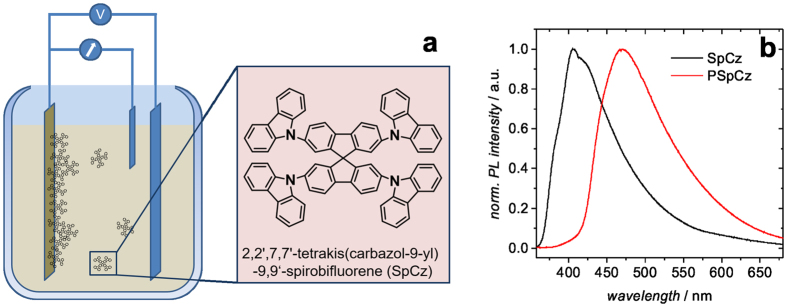
Principle of electrochemical polymerization into a microporous network PSpCz from a solution of the monomer SpCz (**a**). Photoluminescence (PL) spectra of the resulting microporous PSpCz in comparison to the PL of a thermally evaporated thin film of the monomer (SpCz).

Figure 1a shows a schematic of the electrochemical polymerization of SpCz monomers to microporous films of PSpCz. The substrate is ITO coated glass which forms the working electrode. Further details of the electrochemical polymerization process can be found in the experimental section and in a previous report^10^. Please note, in a comparative study published earlier, PSpCz has shown the highest BET surface area (1300 m^2^/g) compared to other microporous carbazole-based compounds prepared in a similar fashion. The photo-luminescence (PL) spectrum of PSpCz in comparison to that of a thermally evaporated SpCz thin film is shown in Fig. 1b.

As expected, the PL maximum of the polymerized network (*λ* = 472 nm) is substantially red-shifted compared to the monomer film (*λ* = 407 nm). A similar red-shift has been observed for other microporous polymer structures with respect to films of their corresponding monomers^12^,^15^. The absorption spectrum of the monomer SpCz shows a sharp onset at 378 nm (3.28 eV), while in the polymer the main absorption edge can be found red-shifted at 420 nm (2.95 eV) (Figure S1). This red-shift is in agreement with the shift found in the PL spectra of SpCz and PSpCz. Moreover, the absorption spectrum of PSpCz exhibits a pronounced low energy tail extending up to about 550 nm, pointing to some significant disorder in the polymerized material. The HOMO levels for both PSpCz and SpCz have been determined by atmospheric pressure photo-electron spectroscopy to HOMO_SpCz_ = −5.83 eV and HOMO_PSpCz_ = −5.58 eV (Figure S2). The HOMO level of the monomer SpCz is in agreement with previous reports for the HOMO levels of similar carbazole compounds, i.e. −5.63…−6.2 eV for 4,4′-Bis(N-carbazolyl)-1,1-biphenyl (CBP)^16^,^17^,^18^ or −5.82 eV for 4,4′,4″-tris(N-carbazolyl)triphenylamine (TCTA)^19^. Note, the cross linking in the PSpCz occurs via its carbazole units. The difference in the HOMO levels of the monomer and the polymerized film is therefore reasonable, as the HOMO of many carbazole-based compounds, e.g. CBP, has been shown to be predominantly governed by the carbazole units^17^. For an application as luminescence sensor the optical gap of the respective material is of paramount importance. It has been shown that the bandgap of organic materials determined by photo-emission may substantially deviate from the so-called optical gap (*E*_opt_), which is relevant for the absorption/emission of photons^20^,^21^. *E*_opt_ can be estimated from the short-wavelength onset of the PL spectrum. From the PL spectra of SpCz and PSpCz (Fig. 1b), *E*_opt_ = 3.4 eV and *E*_opt_ = 3.0 eV is derived, respectively. Thus, an estimate of the LUMO position based on the data for *E*_opt_ results in positions of the LUMO levels as LUMO_SpCz_ = −2.43 eV and LUMO_PSpCz_ = −2.58 eV. This is somewhat deeper than the LUMO levels of CBP and TCTA, which have been determined by inverse photo emission or electrochemical methods to be on the order of −2.2 eV^16^,^17^,^18^,^19^.

AFM images of nominally 50 nm thick films of thermally evaporated SpCz and electrochemically deposited microporous PSpCz are shown in the supporting information (Figure S3). The roughness is 2.6 nm (rms) for the SpCz layer and it increases significantly to 14 nm (rms) for the microporous PSpCz. In an attempt to estimate the pore size in the MPN films, we assumed a model substance with spherical pores, which for simplicity are arranged in a regular simple cubic structure (Figure S4). With a BET surface of 1300 m^2^/g and an assumption of a bulk mass density of the non-porous polymer of 1.2 g/cm^3^, a typical density for a wide range of organic thin films^22^, an upper limit for the pore diameter of 4 nm can be derived. Given the simplicity of the estimate, this result is in reasonable agreement with previous reports of pore diameters on the order of 2 nm for carbazole based electropolymerized MPNs^12^,^23^.

**Figure 2:**
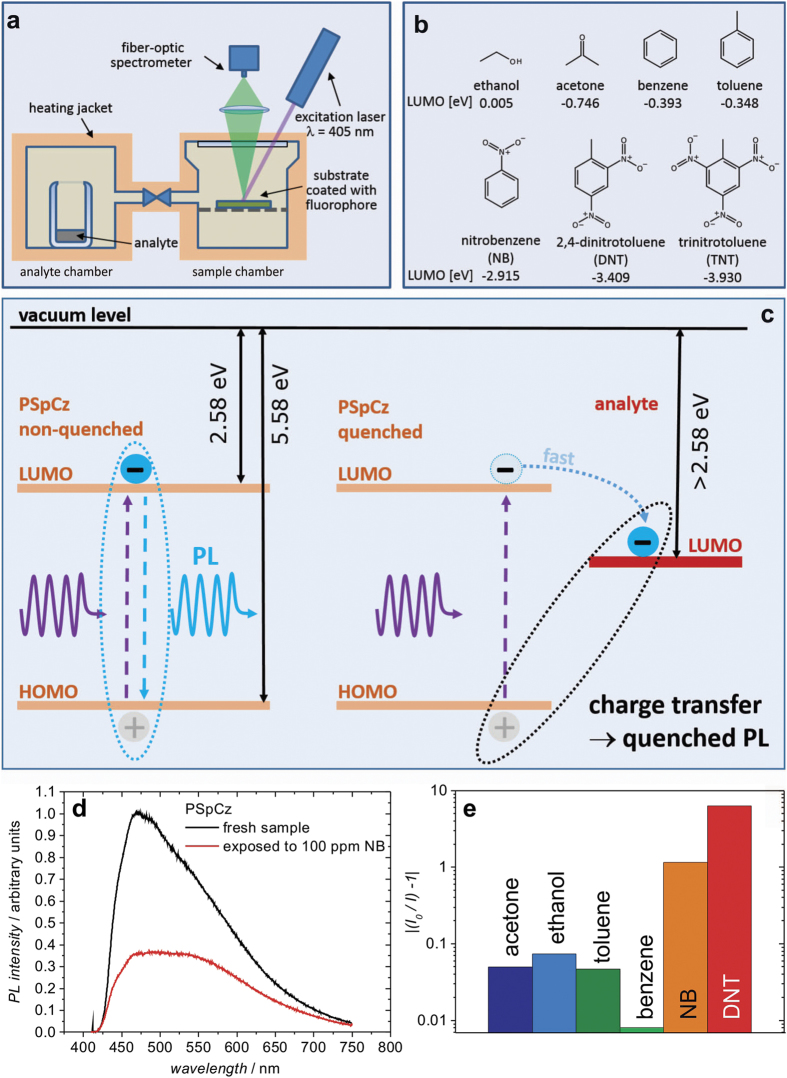
Setup for the characterization of the photoluminescence of the sensors upon controlled exposure levels of various analytes (**a**). Molecular structures of the analytes considered in this study, along with their reported LUMO level positions^24^ (**b**). Principle of luminescence sensors: Energy level scheme of PSpCz and fate of an exciton without and with quenching analyte molecule. (**c**) Example of the PL spectra of PSpCz in the fresh state and after exposure to 100 ppm of NB (**d**). Relative change of the integrated PL intensity of the luminescent sensor before (*I*_0_) and after (*I*) exposure to 100 ppm of the respective analyte (**e**). Note, the absolute value of the quenching efficiency, |*I*_0_/*I* − 1|, is plotted.

In order to assess the sensor properties of the microporous PSpCz layers, they have been exposed to vapors of various analytes. For this purpose a setup as that shown in Fig. 2a has been used. Specifically, the analyte is positioned in a separate chamber (analyte chamber), which is connected to the chamber where the PSpCz coated substrate is located (sample chamber). The entire system can be heated.

Details about the measurement setup can be found in the experimental section. In an orienting experiment, which was aimed to check for the selectivity of the PSpCz as luminscence sensor, we used the analytes shown in Fig. 2b. As an example, Fig. 2d shows the decay of the PL spectrum of PSpCz upon exposure to 100 ppm of nitrobenzene (NB) for 2.5 min. For a comparison of the PL response of the sample to various analytes, a so-called quenching efficiency (*I*_*0*_/*I*) −1 can be defined, where *I*_0_ and *I* are the integrated PL intensities of the luminescent sensor before and after exposure to the analyte, respectively.

The quenching efficiency of PSpCz upon exposure to 100 ppm of various analytes is shown in Fig. 2e. Please note the logarithmic representation of the vertical axis. Obviously, a significant quenching efficiency >1 is found in the case of electron deficient nitro-aromatic analytes (NB, DNT) with a LUMO level below that of PSpCz (LUMO_PSpCz_ = −2.58 eV), while for the other analytes (with significantly higher LUMO levels) only a negligible effect on the PL is observed.

The mechanism of PL quenching, which is schematically shown in Fig. 2c, is therefore in line with an electron transfer quenching mechanism proposed earlier^14^. As a result, the higher quenching efficiency of DNT compared to NB can be attributed to the significantly deeper LUMO level of DNT compared to NB, providing more driving force for the electron transfer from the LUMO of PSpCz to the LUMO of DNT. Note, as we were not able to provide 100 ppm of TNT due to its low vapour pressure, we did not include TNT in this comparison. The response of PSpCz to TNT will be discussed in detail below. As shown in Figure S5, the PL quenching is reversible by removing the analyte from the MPN. Please note, most gaseous species which are potentially present in our atmosphere, e.g. O_2_ (*E*_A_ = 0.45 eV), O_3_ (2.1 eV), NO_2_ (2.27 eV), SO (1.12 eV), SO_2_ (1.1 eV), H_2_O (0.9–1.3 eV) etc., show electron affinities in the range of 0.5–2.3 eV 25, which would not be expected to lead to an electron transfer from the LUMO of the PSpCz (*E*_A_ = 2.58 eV).

**Figure 3:**
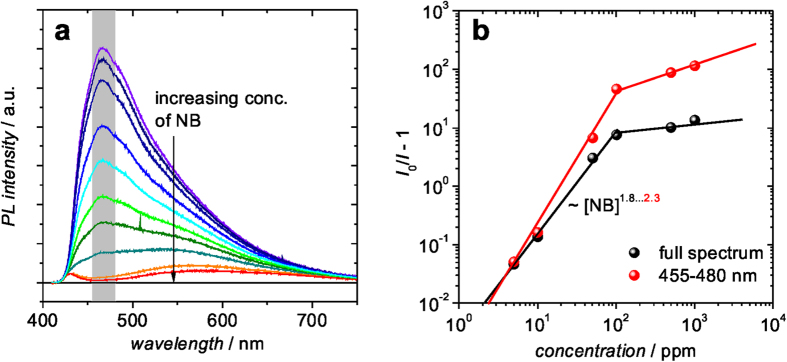
PL spectra of PSpCz upon exposure to various concentrations of nitrobenzene ranging from no exposure (violet) to 1000 ppm (red) (**a**), and the resulting plot of the quenching efficiency vs. concentration of NB (**b**). For the determination of *I* and *I*_0_ either the full spectrum or the spectral region between 455−80 nm (marked in (**a**)) has been chosen.

In Fig. 3 the quenching efficiency upon variation of the analyte concentration of nitrobenzene is studied. The quenched PL spectra are shown in Fig. 3a. The resulting quenching efficiency (I0/I − 1) for a concentration varied over several orders of magnitude is shown in Fig. 3b. Here, we have either used the integrated intensity of the full PL spectra or of a selected spectral region (455–480 nm) around the spectral maximum. Obviously, a somewhat higher quenching efficiency is determined if only the spectral region around the PL maximum is considered. This result may be explained by the fact that excitons belonging to the low-energy tail of the PSpCz spectrum (λ > 550 nm ⇔ hν < 2.25 eV) may not experience enough driving force for dissociation by electron transfer from the PSpCz to the LUMO of the NB located at −2.915 eV. Note, the energy difference between HOMOPSpCz = −5.58 eV and that of a photon with hν = 2.25 eV is about −3.3 eV, which would be below the LUMO of nitrobenzene. Thus, for a transfer from PSpCz to NB, electrons in this case would have to go uphill in energy. As a result, the PL spectra in the thoroughly quenched state of the PSpCz show a residual spectral feature in the low-energy region. Note, some significant signature of low energy states can be seen in the absorption spectrum of PSpCz (Figure S1).Interestingly, as shown in the log-log plot of Fig. 3b, in the regime of low concentrations of nitrobenzene ([NB]), a non-linear behaviour for the quenching efficiency is found with (*I*_*0*_*/I* − 1) ∼ [NB]^x^, where *x* varies between 1.8–2.3. It has to be noted that in the ideal Stern-Volmer model (*I*_*0*_/*I* − 1) is proportional to the concentration of the quencher (i.e. *x* = 1). The prerequisites for the linearity are the equal accessibility of the quenching molecules to all luminescent parts of the sensor and the prevalence of only one quenching mechanism. The origin of the non-linearity in our case is not fully clarified, and has to be the subject of further studies. In general, non-linear effects have been encountered in case of a mix of static and dynamic interaction of chromophore and analyte^26^.

At elevated concentrations of the analyte ([NB] > 100 ppm), the quenching efficiency levels off and saturates in a range of 10–100. Again the evaluation of the spectral region around the PL maximum shows a higher saturation quenching efficiency, possibly due to the reasons discussed above. The origin of this saturation behavior could be the full penetration of the analyte into the PSpCz pores and saturated quenching of all fluorophores which experience sufficient energetic driving force for electron transfer.

NB and DNT are both molecules with a relatively high vapor pressure (0.25 mbar and 1.9 × 10^−4^ mbar) even at room temperature^27^,^28^. Opposed to that, TNT owing to is low vapor pressure is substantially more challenging to detect in the gas phase. To explore the detection limits of our PSpCz MPN layers for nitro-aromatic vapors, we studied its response to TNT.

For the control of the vapor pressure of the TNT analyte, we have used the heating capability of our measurement setup, as shown in Fig. 2a. The relation of TNT vapor pressure and temperature has been estimated according to the Clausius-Clapeyron-equation with parameters as reported in the literature, i.e. log_10_(*P*) = *A* − *B*/*T*, where *P* is the pressure in Torr and *T* is the temperature in Kelvin (*A* = 14.74, *B* = 5960)^28^.

**Figure 4:**
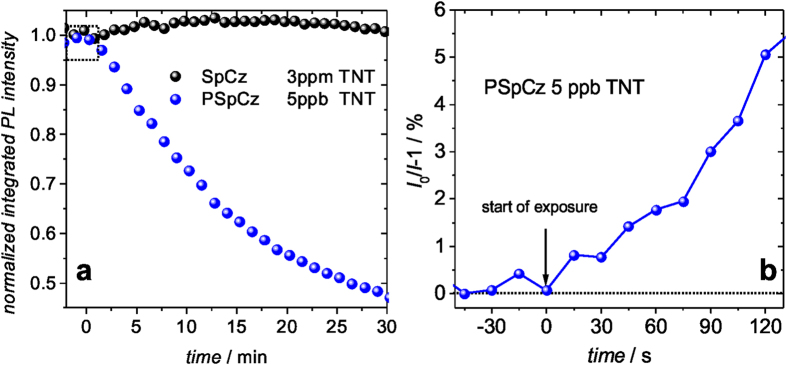
Comparison of the response of the integrated PL intensity of SpCz and PSpCz to different levels of TNT exposure (**a**). Quenching efficiency of PSpCz exposed to 5 ppb at short time scales. Exposure to TNT starts at *t* = 0 s (**b**).

For comparison, we have studied the PL response of a SpCz thin film prepared by thermal evaporation and that of a microporous PSpCz layer. Both were nominally 50 nm thick, but while the thermally evaporated layer is rather dense with a BET surface area of only 0.88 m2/g, the microporous network in the PSpCz affords a surface area of 1300 m2/g. Therefore, we assume that the interaction of TNT and SpCz happens predominantly at the surface of the SpCz layer. At a TNT concentration of 3 ppm, no detectable PL quenching effect in SpCz can be observed even within 30 min of exposure (Fig. 4a). This indicates that the integrated intensity of the quenched PL at the surface of the SpCz film relative to the non-quenched PL of the volume of the layer is below the detection limit of the setup. In strong contrast, the microporous PSpCz clearly shows a detectable PL quenching even at three orders of magnitude lower TNT concentrations of 5 ppb, roughly corresponding to the vapor pressure of TNT at room temperature (22 °C). This detection level impressively demonstrates the importance of the microporous morphology, which facilitates the accessibility of the TNT molecules to a substantially larger amount of chromophore units compared to the case of the thermally evaporated (non-porous) thin film of SpCz. Figure 4b shows that even on a time scale of seconds, a clearly detectable quenching efficiency can be found for a non-optimized PSpCz sample exposed to 5 ppb of TNT. The limits of various electron rich chromophore systems for the detection of TNT and other nitro-aromatic compounds has been recently reviewed29. The ability to detect levels of TNT in the gas phase equivalent to the vapor pressure at room temperature, puts our non-optimized microporous PSpCz on the same level with the most sensitive luminescence sensors for nitro-aromatics. We want to note that the detection limits of techniques like ion mobility spectroscopy or time of flight mass spectroscopy may reach the sub-ppt level30,31, but at the same time these are significantly more complicated and expensive tools. For comparison, a collection of other sensing techniques can be found in Table S1 (supporting information) and in the comprehensive review by Caygill et al.2.

Future optimization regarding quenching efficiency (further lowered detection limit) and faster response should be possible by designing MPNs with deeper LUMO levels, optimized pore sizes and optimum thickness of the MNP layer.

## Conclusions

In summary, we have shown microporous layers of electrochemically crosslinked spiro-carbazole (PSpCz) as highly sensitive luminescence sensors for vapor-phase traces of nitro-aromatic compounds. Their high BET surface area of 1300 m^2^/g, which is more than three orders of magnitude higher than that of a thermally evaporated thin film of the monomer SpCz (0.88 m^2^/g), provides facile access of analyte molecules. The high LUMO level of PSpCz of −2.58 eV provides a high driving force for electron transfer to electron-deficient nitro-aromatic compounds, like DNT and TNT. In striking contrast to a control sample based on thermally evaporated (non-porous) SpCz, which does not show any luminescence quenching upon exposure to TNT at levels below 3 ppm, the microporous PSpCz shows a clearly detectable response even at TNT concentrations as low as 5 ppb, clearly demonstrating the advantage of microporous films as luminescent sensors for traces of explosive analytes.

## Methods

### Materials synthesis, film deposition

All reagents and chemicals were purchased from commercial sources, unless otherwise stated. ^1^H and ^13^C NMR spectra were obtained on Bruker Avance III 600 machine. APLI mass spectra were recorded on a Bruker Daltronik micrOTOF system (KrF*-Laser ATLEX-SI, ATL Wermelskirchen). Elemental analyses were obtained on a Perkin Elmer 240 B.

### Synthesis of 2,2’,7,7’-tetra(carbazol-9-yl)-9,9’-spirobifluorene (SpCz)

2,2′,7,7′-Tetrabromo-9,9′--spirobifluorene (2.00 g, 3.16 mmol), carbazole (2.55 g, 15.23 mmol), copper(I) iodide (2.90 g, 15.23 mmol), potassium carbonate (5.27 g, 38.14 mmol) and 2,2′-bipyridine (0.24 g, 1.53 mmol) were dissolved in 1,2-dichlorobenzene (50 mL), and the mixture was stirred under argon atmosphere and exclusion of light at 180 °C for 3 days. After filtration through celite and washing with hot toluene, the crude product was obtained by evaporation of the solvents. Further purification by column chromatography on silica, (eluent: hexane/dichloromethane 6:3) and recrystallization from a hexane/dichloromethane mixture ended up in the product as white powder. (Yield: 1.81 g, 83%). ^1^H NMR (600 MHz, C_2_D_2_Cl_4_) δ: 8.16 (d, J = 7.7 Hz, 8H), 8.12 (d, J = 8.1 Hz, 4H), 7.70 (dd, J = 8.0, 1.9 Hz, 4H), 7.46–7.41 (m, 8H), 7.38–7.33 (m, 16H), 7.32 (d, J = 1.8 Hz, 4H). ^13^C NMR (151 MHz, C_2_D_2_Cl_4_) δ: 149.76, 140.57, 140.05, 137.27, 127.27, 126.00, 123.09, 122.47, 121.99, 120.34, 120.08, 109.40, 65.75; MS (APLI) 976.346 [976.357] (M^+^). Ele.Anal. for C_73_H_44_N_4_, found: C 89.79, H 4.39, N 5.73, calc: C 89.73, H 4.54, N 5.73.

### Electrochemical polymerization of SpCz on ITO

Acetonitrile and dichloromethane (HPLC grade) were refluxed over phosphorus pentoxide for 3 h and distilled. Tetrabutylammonium perchlorate (TBAP, for electrochemical analysis, ≥99.0%) was purchased from Sigma-Aldrich. Indium tin oxide-coated transparent electrodes on glass (ITO, R_sh_ ≤ 20 Ohm/sq.) were purchased from Präzisions Glas & Optik GmbH.

For electrochemical polymerization, 10 mL of 0.5 mM SpCz were prepared in acetonitrile/dichloromethane (1:4) mixture using 0.1 M TBAP as supporting electrolyte. The solutions were placed in a three-electrode cell connected to a Potentiostat/Galvanostat PAR VersaSTAT 4 under argon atmosphere at 25 °C. ITO (~1.5 × 1.2 cm deposit area) on glass and a platinum gauze (2.5 × 1.2 cm area), separated by 1  cm, were used as WE and CE, respectively. Ag°/AgNO_3_ (0.1 M AgNO_3_, 0.60 V vs NHE) was used as reference electrode (RE). Thin films of PSpCz were obtained by applying 10 cyclic voltammograms from 0 to 0.98 V with a scan rate of 0.1 Vs^**−**1^ (Figure S6). For krypton gas sorption isotherms, thick films were produced by applying an oxidative potential of 1 V for 20 min. A potential of 0 V was applied after the polymerization for 60 s in order to discharge the deposits. After rinsing the deposits with acetonitrile and dichloromethane, they were dried for 20 min at 85 °C.

As control samples thin films of the monomer SpCz were thermally evaporated in high-vacuum (10^−7^ mbar). The layer thickness was 50 nm as controlled by a stylus profiler. For a reasonable comparison with the PSpCz samples, we have used glass/ITO substrates for the thermally evaporated SpCz thin films.

### Materials characterization

Krypton sorption isotherms were recorded on a BEL Japan Inc. Belsorp-max system at 77 K. Samples were dried on a Belprep-vac II at 140 °C and ~2 Pa overnight prior to the gas sorption measurements. Optical absorption was measured using a JASCO V-670 UV-VIS spectrometer. The ionization energy of SpCz and PSpCz was determined by atmospheric pressure photoelectron spectroscopy using a Riken Keiki AC-2.

For determining transmission and reflection spectra a Deuterium Halogen lamp (DH-2000-BAL, OceanOptics) and a spectrometer with a range from 186 nm to 1041 nm (USB 2000 + XR1-ES) were used.

The photoluminescence (PL) of the samples was measured in the setup shown in Fig. 2a. For excitation, a diode pumped solid state laser (λ = 355 nm, 11 mW/cm^2^) or a UV laser diode (λ = 405 nm, 35 mW/cm^2^) was used. The PL signal was coupled into a monochromator and detected by a cooled charge coupled device camera (Princeton Instruments).

### Luminescence quenching experiments

The apparatus used in the quenching experiments is a home-built system of two stainless steel chambers connected by a needle valve. The temperature in the chambers can be controlled separately by heaters. One of the chambers contains the analyte, while the other chamber contains the substrate with the sensor film. This chamber is equipped with an optical port for PL measurement. For the high vapour pressure analytes, their concentrations in the sample were adjusted by the needle valve between the two chambers monitored by a vacuum gauge. For analytes with limited vapour pressure, e.g. TNT, the vapour pressure in the experiment has been controlled by the temperature of the analyte chamber. Note, the temperature of the sample chamber was always kept somewhat higher than that of the analyte chamber to avoid condensation.

## Additional Information

**How to cite this article**: Räupke, A. *et al*. Highly sensitive gas-phase explosive detection by luminescent microporous polymer networks. *Sci. Rep*. **6**, 29118; doi: 10.1038/srep29118 (2016).

## Supplementary Material

Supplementary Information
