# Supplementary material for: Highly sensitive gas-phase explosive detection by luminescent microporous polymer networks
Source: Sci Rep. 2016 Jul 4;6:29118. doi: 10.1038/srep29118 (PMC4931441; doi:10.1038/srep29118)
Supplement: Supplementary Information [file srep29118-s1.pdf]

Supplementary Information

Highly sensitive gas-phase explosive detection by luminescent microporous polymer networks

André Räupke[a], Alex Palma-Cando[b], Eugen Shkura[a], Peter Teckhausen[c], Andreas Polywka[c], Patrick Görrn[c], Ullrich Scherf[b], and Thomas Riedl[a*]

[a] Institute of Electronic Devices, University of Wuppertal, Rainer-Gruenter-Str. 21, 42119 Wuppertal, Germany

[b] University of Wuppertal, Macromolecular Chemistry, Gaußstr. 20, 42119 Wuppertal, Germany

[c] University of Wuppertal, Chair of Large Area Optoelectronics, Rainer-Gruenter-Str. 21, 42119 Wuppertal, Germany

E-mail: t.riedl@uni-wuppertal.de

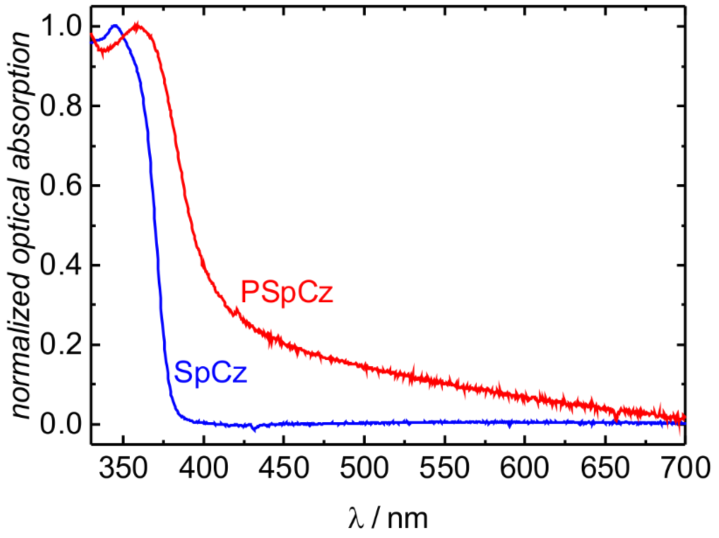

Figure S1 Optical absorption spectra of SpCz and PSpCz.

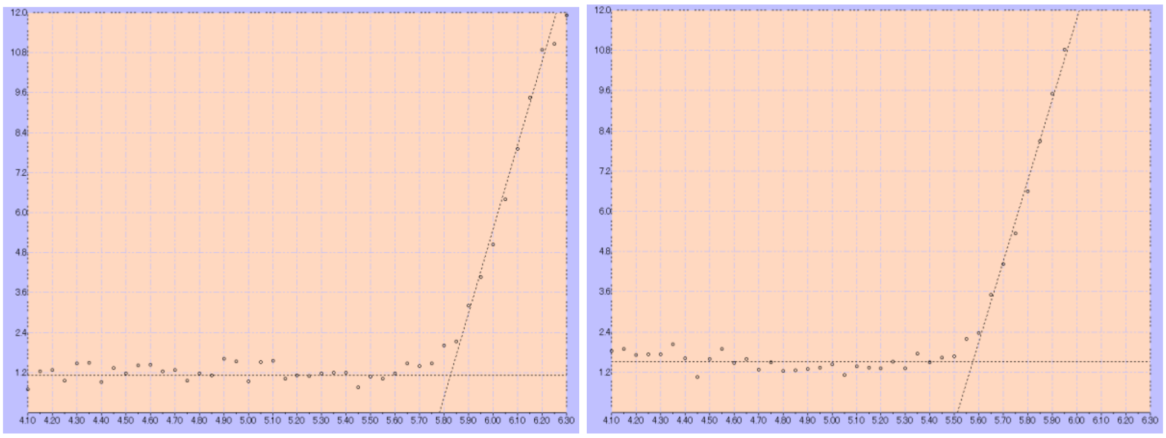

Figure S2 Photoemission spectra for SpCz (left) and PSpCz (right).

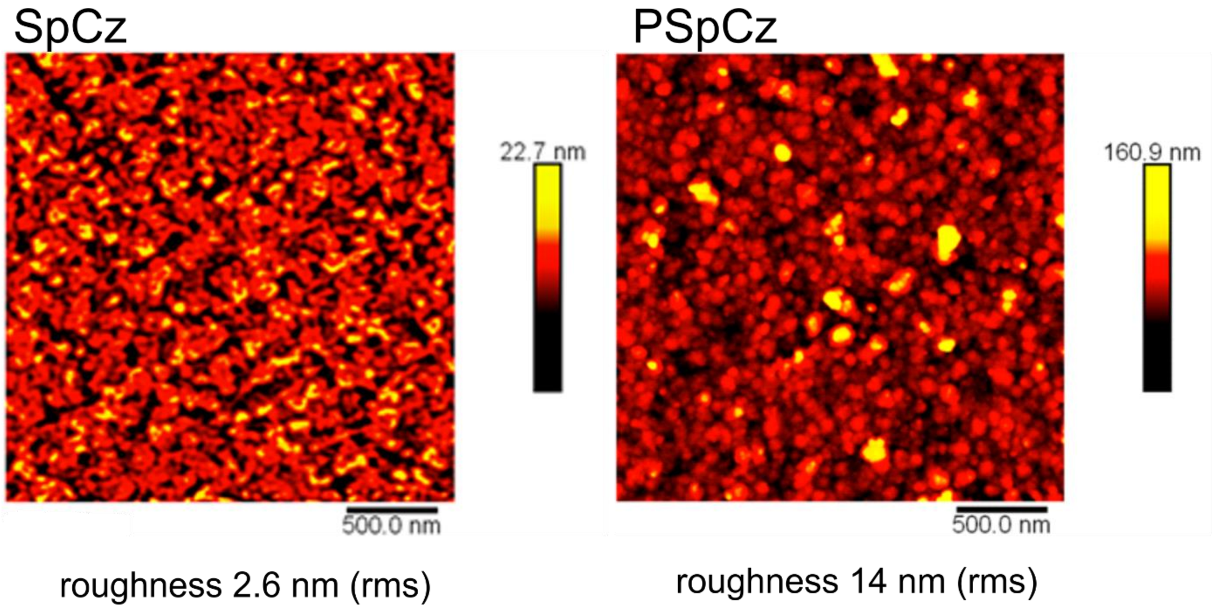

Figure S3 Atomic force microscopy images of thermally evaporated SpCz (left) and PSpCz (right).

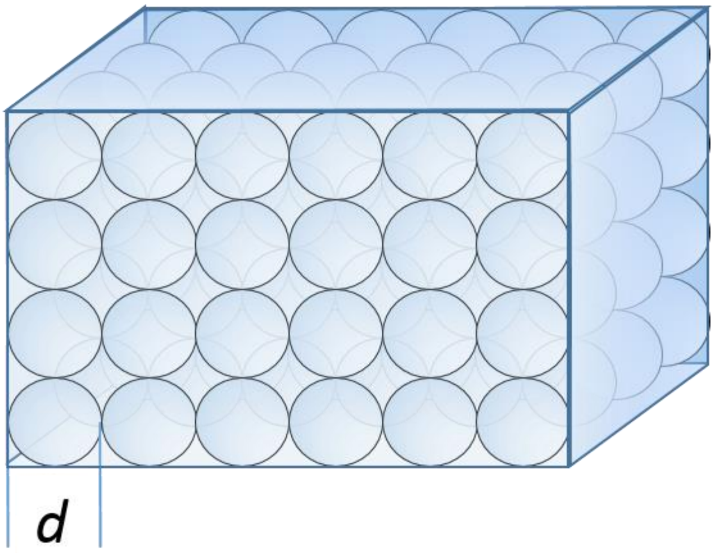

Figure S4 Simple spherical pore model to estimate the pore diameter of the MPN PSpCz films. The pores are assumed to be arranged in a simple cubic structure.

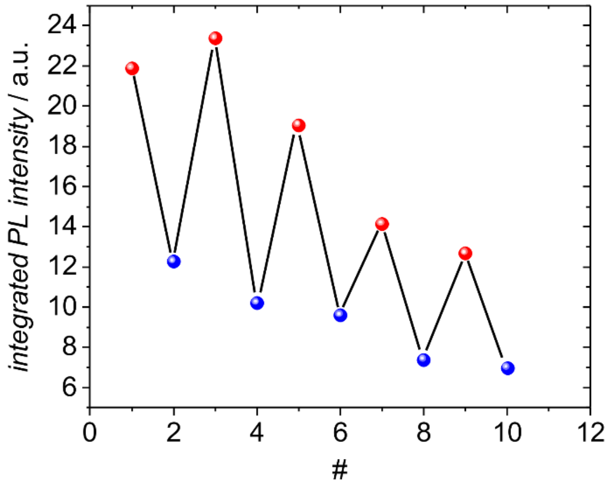

Figure S5 Integrated PL intensity of a PSpCz sensor before (red) and after (blue) repeated exposure to 100 ppm of nitrobenzene. Note after each exposure the sensor has been washed in warm (80°C) toluene for one hour to remove residues of the NB analyte. While the quenching is fully reversible upon the first washing, some degradation of the sensor layers is seen by an overall drop of the PL intensity upon repeated washing steps. Some damage due to the toluene cannot be excluded.

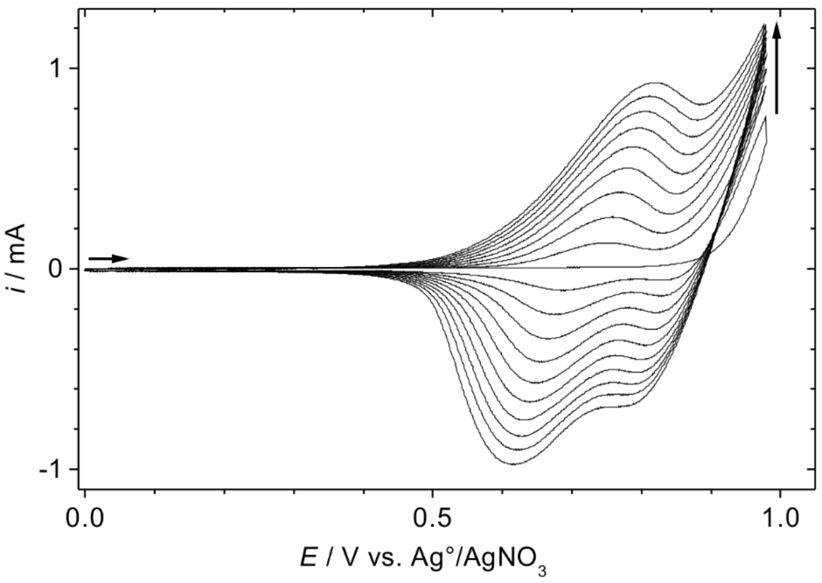

Figure S6 Ten consecutive cyclic voltammograms for electropolymerization of 0.5 mM SpCz in acetonitrile/dichloromethane (1:4) mixture on ITO, potential range: 0 to 0.98 V, scan rate: 0.10 Vs^{-1}, 0.1 M TBAP as supporting electrolyte.

Table S1 Various detection principles for nitro-aromatic compounds.

detection principle	analyte	concentration	Ref.
ion mobility spectroscopy	TNT	< ppt	[1]
ToF mass spectroscopy	TNT	0.3 ppt	[2]
cavity ring down spectroscopy, laser at 6-8 µm wavelength	TNT	75 ppt	[3]
surface acoustic wave sensors	TNT	50 ppb	[4]
µ gas chromatography	DNT	100 ppt	[5]
surface enhanced raman spectroscopy	DNT	5 ppb	[6]
micro cantilever	TNT	100 ppt	[7]
chemiluminescence	TNT	34 ppb	[8]
quartz crystal microbalance	TNT	100 ppb	[9]

1 Ewing, R. G., Atkinson, D. A., Eiceman, G. A. & Ewing, G. J. A critical review of ion mobility spectrometry for the detection of explosives and explosive related compounds. *Talanta* **54**, 515-529, doi:10.1016/S0039-9140(00)00565-8 (2001).

2 Mullen, C. *et al.* Detection of Explosives and Explosives-Related Compounds by Single Photon Laser Ionization Time-of-Flight Mass Spectrometry. *Anal Chem* **78**, 3807-3814, doi:10.1021/ac060190h (2006).

3 Todd, M. W. *et al.* Application of mid-infrared cavity-ringdown spectroscopy to trace explosives vapor detection using a broadly tunable (6–8 μm) optical parametric oscillator. *Applied Physics B* **75**, 367-376, doi:10.1007/s00340-002-0991-8 (2002).

4 Kapoor, J. C. & Kannan, G. K. Landmine Detection Technologies to TraceExplosive Vapour Detection Techniques. *2007* **57**, 14, doi:10.14429/dsj.57.1818 (2007).

5 Collin, W. R. *et al.* Microfabricated Gas Chromatograph for Rapid, Trace-Level Determinations of Gas-Phase Explosive Marker Compounds. *Anal Chem* **86**, 655-663, doi:10.1021/ac402961t (2014).

6 Sylvia, J. M., Janni, J. A., Klein, J. D. & Spencer, K. M. Surface-Enhanced Raman Detection of 2,4-Dinitrotoluene Impurity Vapor as a Marker To Locate Landmines. *Anal Chem* **72**, 5834-5840, doi:10.1021/ac0006573 (2000).

7 Yang, Y., Chen, Y., Xu, P. & Li, X. Quad-cantilever microsensors with a low-cost single-sided micro-machining technique for trace chemical vapor detection. *Microelectron Eng* **87**, 2317-2322, doi:10.1016/j.mee.2010.03.010 (2010).

8 Monterola, M. P. P., Smith, B. W., Omenetto, N. & Winefordner, J. D. Photofragmentation of nitro-based explosives with chemiluminescence detection. *Anal Bioanal Chem* **391**, 2617-2626, doi:10.1007/s00216-008-2177-7 (2008).

9 Larsson, A., Angbrant, J., Ekeroth, J., Månsson, P. & Liedberg, B. A novel biochip technology for detection of explosives – TNT: Synthesis, characterisation and application. *Sensors and Actuators B: Chemical* **113**, 730-748, doi:10.1016/j.snb.2005.07.025 (2006).
